# Severe subacute combined degeneration of the spinal cord resulting from nitrous oxide (N2O) abuse: a case series

**DOI:** 10.1186/s42466-024-00364-x

**Published:** 2025-02-13

**Authors:** Lucas C. Adam, Anuschka Grobelny, Katrin Hahn, Heinrich J. Audebert, Patricia Krause, Christiana Franke, Klemens Ruprecht

**Affiliations:** 1 Department of Neurology and Experimental Neurology, Charité - Universitätsmedizin Berlin, corporate member of Freie Universität Berlin and Humboldt-Universität zu Berlin, Hindenburgdamm 30, 12203 Berlin, Germany; 2 Department of Neuroradiology, Charité - Universitätsmedizin Berlin, Freie Universität Berlin and Humboldt-Universität zu Berlin, Charitéplatz 1, 10117 Berlin, Germany

**Keywords:** Vitamin B12 deficiency, Nitrous oxide (N2O), Myelopathy, Neurotoxicology, Subacute combined degeneration of the spinal cord (SCD, SACD), Funicular myelosis

## Abstract

**Objective:**

To describe the demographic, clinical, laboratory, and radiological findings, and the clinical course of seven patients with severe N2O-induced subacute combined degeneration of the spinal cord (SACD).

**Methods:**

Retrospective study with prospective follow-up of patients with SACD associated with N2O abuse presenting at a single center between 2014 and 2024.

**Results:**

The median age (range) of the seven patients (one woman, six men) was 24 (18–33) years. Prior to disease onset, patients had consumed N2O daily over a median (range) of 12 (3-20) weeks, with a mean (SD; range) inhalation dosage of 2376.7 (2872.7; 160–9000) g of N2O per day. Clinical presentations included paresthesia and paresis in the legs and gait disturbances. All patients exhibited characteristic signal alterations in the posterior columns spanning from C1 to T10 on T2-weighted spinal MRIs. Electrophysiology demonstrated polyneuropathies in all but one patient. Vitamin B12 levels were decreased in four, but normal in three patients. Methylmalonic acid levels were elevated in all patients. Although the median (interquartile range [IQR]) modified Rankin Scale score improved from 3.0 (3.0–4.0) at baseline to 1.0 (1.0–2.0; *p* < 0.05, Wilcoxon matched-pairs signed-rank test) at follow-up after the start of vitamin B12 supplementation, all five patients who could be examined on follow-up exhibited persistent deficits on the last follow-up assessment at a median (range) of 5 (3-116) months after disease onset.

**Conclusions:**

N2O abuse over a few weeks can lead to severe SACD. The diagnosis is supported by characteristic findings on spinal MRI and elevated methylmalonic acid levels, while normal vitamin B12 levels do not rule out N2O-induced SACD. Although there was some clinical improvement upon cessation of N2O abuse and vitamin B12 supplementation, residual deficits persisted.

## Introduction

Nitrous oxide (N2O) is a non-flammable gas, first described and synthesized in 1772 by the English chemist Joseph Priestley [1]. Nowadays, N2O is widely used as an anesthetic agent, particularly in minor surgical procedures and dental treatments. It is also used by the food industry, notably in the production of whipped cream and as a propellant in aerosol cans [2].

Due to its euphoric properties when inhaled, N2O has formerly been misused at “laughing gas parties” during the 19th century, and more recently in modern club and music scenes. Its low cost and easy accessibility have facilitated its increasing global recreational consumption [3]. In response, European countries such as France and the United Kingdom, have recently regulated or banned the unrestricted sale of N2O [4]. In Germany, the consumption and sale of N2O remain unregulated to date [5].

Although precise data on the prevalence of N2O consumption in Germany is lacking, there is evidence suggesting a significant increase in usage in recent years [6]. N2O is available in various sizes, ranging from small 8 g cartridges intended for culinary use to larger 640 g, 1.25 kg, and 2 kg containers used in medical, industrial, and professional settings. Inhalation of high concentrations of N2O has been associated with a spectrum of neurological and psychiatric symptoms [7] and in particular with subacute combined degeneration of the spinal cord (SACD), also known as funicular myelosis [8]. It is postulated that the pathogenesis of SACD is mediated by a N2O-induced inactivation of vitamin B12 (cobalamin), resulting in functional deficiency. Indeed, N2O irreversibly oxidizes the cobalt ion in cobalamin, rendering it unable to serve as a cofactor for methionine synthase, a critical enzyme in DNA synthesis and myelin production [9].

We here report the demographic, clinical, laboratory, and radiological findings, as well as the clinical course of seven patients diagnosed with severe N2O-induced SACD, collected at a single center between 2014 and 2024.

### Case presentations

Table 1 summarizes the demographic, clinical, and laboratory findings of seven patients with severe SACD treated at the Department of Neurology, Charité – Universitätsmedizin Berlin, between 2014 and 2024. Of note, five of these patients were diagnosed in 2024. The patients’ median (range) age was 24 (18 to 33) years and six out of the seven patients were male. The mean (SD; range) daily inhalation dosage of N2O as reported by the patients was 2376.7 (2872.7; 160–9000) g. The duration of N2O abuse before the onset of the first persisting symptoms varied from 3 to 20 weeks. However, some patients anecdotally reported experiencing acute transient adverse effects, including nausea, vomiting, burning sensations in the mouth and throat, short-lasting cognitive impairment, and pronounced fatigue, shortly after N2O abuse. The patients stated that typical settings of N2O consumption were alone, with friends, or in small groups, rather than in music clubs or at festivals. In addition to N2O, all seven patients also consumed alcohol, tobacco, and at least one further illegal substance, including cannabis, cocaine, 3,4-methylenedioxymethamphetamine (MDMA), opiates, or benzodiazepines. All patients initially presented to the emergency department with newly developed gait disturbances and paresthesia and/or paresis predominantly of the lower extremities. Interestingly, one patient (patient #6) initially presented with sensory disturbances and reduced dexterity of the hands. Disability at initial presentation was moderate to severe, with a median (interquartile range [IQR]) modified Rankin Scale (mRS) score of 3.0 (3.0–4.0, reference range from complete health to death: 0–6). None of the patients followed a vegan or vegetarian diet, had a history of gastric complaints or had other known preconditions for vitamin B12 deficiency.

**Table 1:** Demographic and clinical characteristics of seven patients with N2O-induced SACD

Patient	Age (years)	Sex	Duration of abuse (weeks)	Onset of first symptoms (weeks)	Daily amount of N2O (gram)	Consumption of other drugs	Paresthesia	Pareses	Deep tendon reflexes	Cognitive deficits	Nerve conduction studies
**Patient #1**	19	M	12	12	3200	Cocaine, Cannabis	+++	++	-	+	Sensorimotor mixed demyelinating axonal neuropathy
**Patient #2**	18	M	8	7	1920	Cannabis	+++	++	+	-	Sensorimotor axonal neuropathy
**Patient #3**	24	M	16	12	160	Cannabis, Benzodiazepines, Opiates	+++	-	++	-	Sensorimotor demyelinating neuropathy
**Patient #4**	19	F	24	20	1280	Cannabis	+++	++	+	-	Motor mixed demyelinating axonal neuropathy
**Patient #5**	25	M	8	4	800	Cannabis, MDMA, Cocaine, Benzodiazepines	+++	+	+	+	Motor axonal neuropathy
**Patient #6**	33	M	12	11	277	Cannabis	+++	-	+	+	No signs of neuropathy
**Patient #7**	28	M	4	3	9000	Cannabis, Cocaine	+++	-	+	+	Sensorimotor axonal neuropathy

M: male, F: female, +: mild presence, ++: moderate presence, +++: strong presence, -: not present, n/a: data not available, SACD: Subacute combined degeneration of the spinal cord, MDMA: 3,4-methylenedioxymethamphetamine

**Fig. 1:**
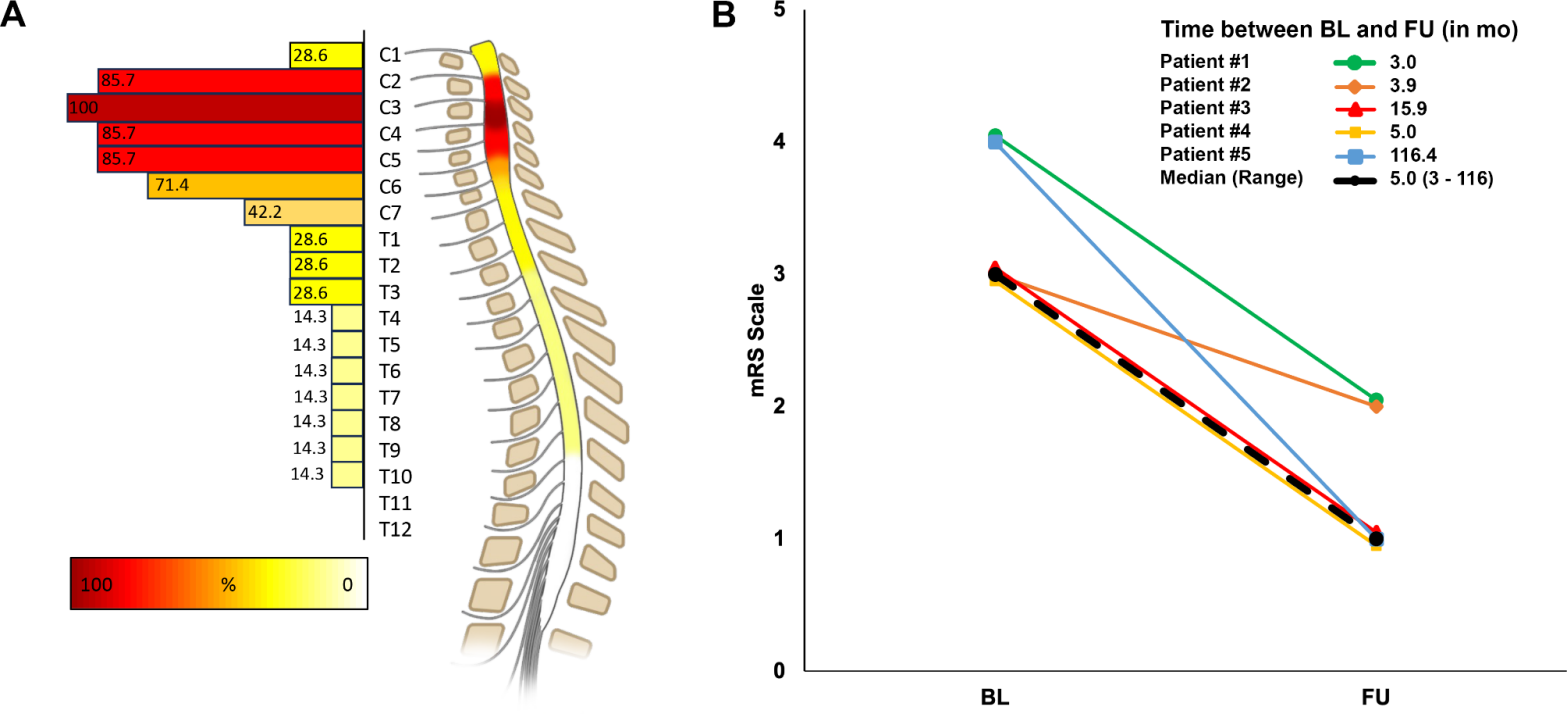
Depiction of the relative frequency (in %) and segment location of SACD in the present case series of seven patients (**A**). The median modified Rankin Scale (mRS) score at baseline (BL) was 3 (IQR: 3–4) and improved to 1 (IQR: 1–2) at follow-up (FU, *p* < 0.05). The FU period ranged from 3 months to 13.5 years. Created with BioRender.com

**Fig. 2:**
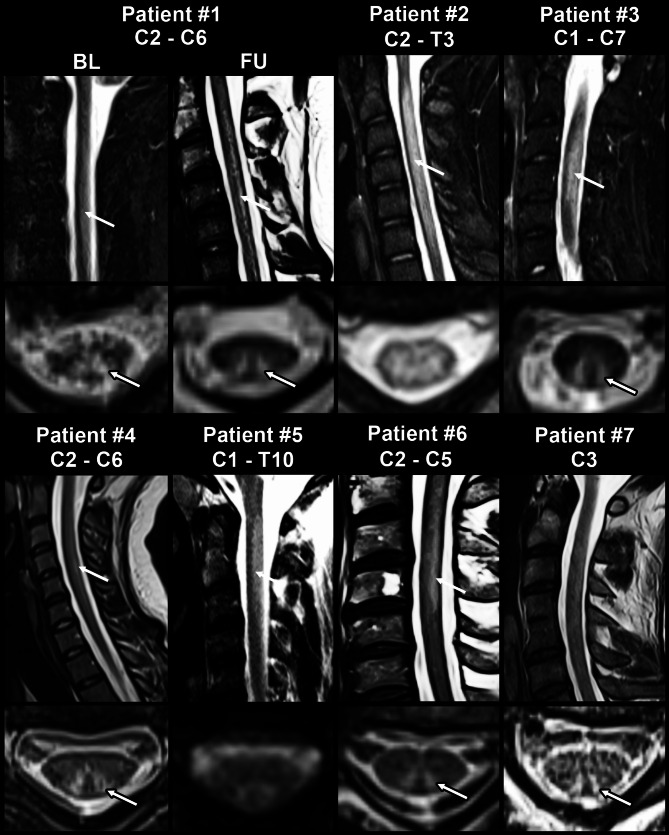
Spinal MRIs of patient #1 – #7 revealed posterior column hyperintensity (white arrow) of the cervical and thoracic spinal cord in T2-weighted midsaggital and T2-weighted axial sections. The extension of the lesion is indicated for each patient, the maximum extension was from C1 to T10. Patient #1, #3, #4, #6 and #7 showed the characteristic “inverted V-sign” on axial sections (white arrow with black contours). Persisting hyperintensities on T2-weighted imaging in patient #1 on follow-up MRI assessment after 4 months. BL: baseline, FU: follow-up

Native MRI of the spinal cord revealed characteristic hyperintensities on T2-weighted sequences in the posterior columns of the cervical and thoracic spinal cord in all patients. Myelopathy signals extended over a median (range) of 5 (1-17) vertebral segments, spanning from C1 to T10, with predominant manifestation in the cervical spinal cord (Figs. 1A and 2). T2-weighted axial sequences revealed an ‘inverted V sign’, a typical lesion pattern of the posterior columns [10], in patients #1, #3, #4, #6, and #7 (Fig. 2).

Nerve conduction studies demonstrated predominantly lower limb sensorimotor polyneuropathies in all patients except for patient #6, with axonal and demyelinating features at varying degrees (Table 1).

Importantly, while four patients had serum vitamin B12 levels below the lower limit of normal (> 191 pg/ml), three patients had normal vitamin B12 levels (Table 2). Among these three patients, one patient (patient #3) had independently initiated vitamin B12 supplementation after conducting online research and another patient (patient #4) had stopped consuming N2O approximately two weeks prior to admission. Nevertheless, functional vitamin B12 deficiency was present in all patients, as indicated by elevated serum levels of homocysteine (5/6 patients), methylmalonic acid (7/7 patients), or holotranscobalamin (2/5 patients). Megaloblastic anemia was observed only in patient #1. Infectious diseases, including HIV and syphilis, were ruled out in five patients. Only one of the patients (patient #5) underwent cerebrospinal fluid examination, which was unremarkable.

**Table 2:** Laboratory findings of seven patients of N2O-induced SACD

Patient	Cobalamin (Vitamin B12) [191–663 ng/l]	Folic Acid [4.6–18.7 µg/l]	Holotranscobalamin [37.5–188 pmol/l]	Methylmalonic acid [< 32 µg/l]	Homocysteine [< 15 µmol/l]	Hemoglobin [13.5–17/12-15.6|	MCV [80–99 fl]	MCH [27–33.5 pg]	Anti-T.pallidum^3^	Anti-HIV 1/2^4^
**Patient #1**	< 100*	12.4	> 300*	368.5*	7.1^2^	11.9*	102.3*	34.4*	Neg	Neg
**Patient #2**	173*	9.5	47.1	713*	n/a	13.6	98.3	32.5	Neg	Neg
**Patient #3**	341	15.9	n/a	119.0*	21.2*	14.6	95.2	33.6*	Neg	Neg
**Patient #4**	332	n/a	58.9	78.0*	20.7*	13.4	94.9	33.8*	n/a	n/a
**Patient #5**	74*	8.8	n/a	15.9 *^1^	21.4*	12.6*	97.0	32.1	Neg	Neg
**Patient #6**	131*	3.5*	63.9	153.5*	49.1*	12.9*	97.0	31.9	Neg	Neg
**Patient #7**	380	10.7	> 300*	205.8*	97.8*	12.8*	90.6	32.3	n/a	n/a

*, abnormal values, n/a: data not available, 1: In patient #5, methylmalonic acid was measured in urine. Reference value for methylmalonic acid in urine is < 5.0 mmol/mol creatinine, 2: collected with a delay of 11 days after vitamin b12 substitution, 3: western immunoblot assay, 4: Electrochemiluminescence (ECL)

All patients were instructed to immediately cease N2O consumption and were promptly treated with vitamin B12 intramuscularly or subcutaneously. In addition, all but one patient were referred to outpatient physiotherapy or rehabilitation. Vitamin B12 treatment was initiated with daily doses of 1000 µg vitamin B12, followed by weekly and later monthly oral administration for 4 to 6 months. One patient (patient #1) was discharged to an inpatient rehabilitation program due to persistent gait disturbances and fine motor skill impairments.

All patients, except for patients #6 and #7, underwent follow-up assessments either in person (*n* = 2) or by phone (*n* = 3) after a median (range) of 5 (3-116) months. All five reassessed patients had only partially recovered and presented with residual symptoms at the last follow-up, including persistent foot and toe drop, distal hypoesthesia and tingling paresthesia. The median (IQR) mRS score improved from 3.0 (3.0–4.0) at baseline to 1.0 (1.0–2.0) at follow-up (*p* < 0.05, Wilcoxon matched-pairs signed-rank test; Fig. 1B). Patients #1 and #2 had follow-up examinations of vitamin B12 metabolites after three and four months, respectively, which showed normalization of vitamin B12 levels. However, patient #2 continued to show persistent functional vitamin B12 deficiency, as indicated by elevated methylmalonic acid and homocysteine levels. Patient #1 underwent nerve conduction studies and an MRI of the cervical spine after 4 months, which showed worsening of the axonal polyneuropathy (data not shown) and overall unchanged T2 hyperintensities, despite clinical improvement (Fig. 2).

## Discussion

Here, we report clinical, laboratory, and radiological findings of seven young adults with N2O-induced SACD. While patients improved following N2O cessation and vitamin B12 substitution, residual symptoms persisted in all five patients assessed on follow-up.

In recent years, an increasing incidence of neurological and psychiatric symptoms associated with N2O abuse has been observed in adults across Europe [11], the United States [12] and Asia [13]. Data from 2014 indicate a lifetime prevalence of N2O use in Germany of 11.2%, with a last-month prevalence of 0.9% [14]. However, more recent data on the prevalence of N2O consumption in Germany are scarce. Nevertheless, the available evidence, primarily from local studies and case reports, suggests a notable increase in N2O usage, particularly among younger people, in recent years [15, 16]. In this respect, it appears remarkable that five of our seven patients were diagnosed during the last year.

All of our patients consumed substances in addition to N2O, potentially indicating a lowered threshold for experimenting with drugs. Physicians should thus actively ask for multi-substance abuse in patients with N2O consumption.

Development of neurological impairments following N2O abuse depends on both the duration and dosage of exposure [17], and may be influenced by individual factors including sex [18], ethnicity [19], gastrointestinal comorbidities, and pre-existing latent vitamin B12 deficiency [20]. The distinct physiological mechanisms underlying N2O-induced myelopathy and neuropathy remain unclear. N2O can cause vitamin B12 deficiency through irreversible oxidization of the cobalt ion in cobalamin, thereby disrupting the methionine and methylation cycles. This disruption impairs critical cellular processes, leading to axonal degeneration and demyelination, particularly within the dorsal and lateral columns of the spinal cord [9]. Additionally, axonal polyneuropathies, another frequently reported N2O-associated neurological condition, appear to be more closely associated with a state of chronic hypoxia resulting from prolonged N2O abuse [21]. However, the minimum quantity of N2O required to reach the threshold for the development of neurological symptoms has not been established [19]. Our present case series shows that only a few weeks of daily N2O abuse can lead to N2O-induced SACD.

The clinical presentations of the patients of our study are consistent with those reported in previous series [19]. Generally, the severity of symptoms appears to be more severe and the speed of their development faster than in patients with vitamin B12 deficiency from other causes.

MR imaging revealed varying degrees of hyperintensity on T2-weighted imaging within the posterior cord of all patients, spanning from C1 to T10. The cervical posterior cord was particularly affected, consistent with findings from a larger British study of 54 patients [19]. This highlights the susceptibility especially of the cervical myelin sheath to vitamin B12 deficiency. While MRI findings can be suggestive of N2O-induced SACD, it is important to note that several other causes of vitamin B12 deficiency, such as a long-term vegetarian diet, digestive system diseases, and gastrointestinal surgery, can present with similar radiologic features [22]. Therefore, a definitive diagnosis of SACD relies on a combination of factors, including a thorough medical history, neurological examination, and the exclusion of other potential causes.

The nerve conduction studies indicated mixed axonal and demyelinating nerve damage. Chronic N2O consumption has been associated with the development of severe peripheral neuropathies, predominantly affecting the lower limbs, characterized by axonal degeneration, demyelination, or a combination of both [23]. A case series with seven patients undergoing electrophysiological follow-up showed decreased, but still present active denervation after short-term follow up accompanied by persistent clinical impairments [24].

Remarkably, three of the seven patients had normal vitamin B12 levels, demonstrating that normal vitamin B12 levels do not rule out the diagnosis of N2O-induced SACD. In line with previous case series, macrocytic anemia was rare [25]. Our findings suggest that homocysteine and methylmalonic acid are reliable indicators of functional vitamin B12 deficiency in patients with N2O-induced SACD. Accordingly, a recent meta-analysis showed that homocysteine and methylmalonic acid levels are elevated in up to 85.0% and 83.3%, respectively, of patients with N2O-induced neurological impairments [7]. Of five patients with available data, only two exhibited abnormal holotranscobalamin levels, suggesting a limited sensitivity in detecting N2O-induced pathologies. Given that holotranscobalamin, the biologically active form of vitamin B12, may not reliably distinguish between functional and dysfunctional forms, its clinical utility as a biomarker for vitamin B12 deficiency in the context of N2O abuse may be limited [26]. Although methylmalonic acid could therefore be a marker of long-term N2O exposure, a thorough evaluation encompassing serum vitamin B12 levels and homocysteine, methylmalonic acid, and holotranscobalamin, might provide the most reliable information on cumulative N2O intake [19].

Despite cessation of N2O abuse and initiation of high-dose vitamin B12 supplementation, all reassessed patients reported persistent residual symptoms at last follow-up. This complies well with findings in patients with N2O-induced SACD from a British cohort, where 89.5% reported symptoms on follow-up [19]. A larger Chinese cohort of recreational N2O abusers with neurological impairments identified female sex as a positive prognostic factor for rehabilitation [18], whereas a French cohort could not identify any significant predictor of long-term outcomes [21].

Unlike in neighboring countries such as France, the Netherlands and Denmark, N2O is available in corner shops and supermarkets in Germany, including urban Berlin, without legal restrictions. Currently, there is an ongoing discussion about regulating the sale of N2O in Germany [5]. Preventive measures and disseminating information appear to be imperative at present, while policy adjustments may be warranted in the future.

Limitations of this study include that data on N2O dosage and duration of abuse were self-reported and could not be independently verified. Furthermore, some of the follow-up assessments were conducted by phone, precluding clinical examination. Nevertheless, all patients presented with severe illness and a strong motivation for medical intervention, reducing the likelihood of intentional misrepresentation. While we cannot exclude that additional use of other drugs may have contributed to the development of SACD, we consider this to be rather unlikely. This hospital-based patient series is biased towards severe cases, potentially underestimating milder N2O-induced neurological impairments. Finally, longer prospective studies are needed to determine the long-term outcomes and identify factors that may facilitate recovery.

In summary, this case series underscores the potentially serious neurological sequelae of N2O abuse in the context unregulated distribution of N2O in Germany. The diagnosis of N2O-induced SACD is supported by characteristic findings on spinal MRI and evidence of functional vitamin B12 deficiency, even in the presence of normal vitamin B12 levels. Early intervention with high-dose vitamin B12 supplementation and physiotherapy can mitigate symptoms, although residual symptoms may persist. Implementing preventive regulations and increasing awareness among young people could potentially reduce the incidence of N2O-induced adverse effects.

## Data Availability

The original contributions presented in the case series are included in the article, further inquiries can be directed to the corresponding author.
